# Features extraction and fusion by attention mechanism for software defect prediction

**DOI:** 10.1371/journal.pone.0320808

**Published:** 2025-04-14

**Authors:** Shaoming Qiu, Bicong E, Jingjie He

**Affiliations:** School of Information Engineering, Dalian University, Dalian, China; IIIT Kurnool: Indian Institute of Information Technology Design and Manufacturing Kurnool, INDIA

## Abstract

Software defect prediction is a technology that uses known software information to predict defects in the target software. Generally, models are built using features such as software metrics, semantic information, and software networks. However, due to the complex software structure and the small number of samples, without effective feature representation and feature extraction methods, it is impossible to fully utilize software features, which can easily lead to misjudgments and reduced performance. In addition, a single feature cannot fully characterize the software structure. Therefore, this research proposes a new method to efficiently and accurately represent the Abstract Syntax Tree(AST) and a model called MFA(Multi Features Attention) that uses a deformable attention mechanism to extract features and uses a self-attention mechanism to fuse semantic and network features. By selecting 21 Java projects and comparing them with multiple models for cross-version and cross-project experiments, the experiments show that the average ACC, F1, AUC of the proposed model in the cross-version scheme reach 0.7, 0.614 and 0.711. In the cross-project scheme, the average ACC, F1 and AUC are 0.687, 0.575 and 0.696. Up to 41% better than other models, and the results of fusion features are better than those of a single feature, showing that MFA using two features for extraction and fusion has greater advantages in prediction performance.

## Introduction

Software defect prediction generally uses three types of features: software metrics, semantic features, and network features. Software metrics mainly refer to hand-crafted features, such as McCabe and CK that measure software structures. In this case, a file often has a fixed value, so it has low robustness and poor model transferability in experimental scenarios such as cross-version and cross-project. Using semantic features and network features often has better performance, but generally requires corresponding deep learning technology processing [1].

Among all the semantic features, such as Source Code, Control Flow Graph(CFG), Abstract Syntax Tree(AST) is widely used [2]. As a pre-check method for compilers, it can eliminate syntax problems in source code and increase the accuracy of semantic features. The common method for processing semantic features using AST is to traverse the AST, then either perform word embedding on the traversal sequence or represent it as an integer sequence, which is input into the model for learning. However, the tree structure of AST cannot be effectively represented before deep learning, which can easily cause semantic feature loss.

Some studies [3,4] introduce CFG to supplement the missing structures in AST traversal sequences or treat AST as graphs. They generally use graph embedding or neural networks similar to GNN to process graph structure, which not only increases the complexity of feature representation but also involves a considerable amount of hyperparameters and workload. Other studies tend to optimize the AST structure to reduce its complexity. Quantification or decomposition of the AST is a common approach. Munir et al. [5] designed 32 types of statement-level metrics to measure the AST structure. Jiang et al. [6] pruned the AST to reduce the depth. These methods have increased the model’s processing capability and learning speed, but they have also compromised the integrity of the AST structure, resulting in bias.

Semantic features are only one aspect of the software. They primarily define the software’s functionality and determine the usability of a particular module. Software is an interconnection and combination of multiple modules; thus, it is also necessary to consider the relationships between the modules to achieve the ideal goal of high cohesion and low coupling. The software network, as a supplement to AST, is receiving increasing attention from researchers.

So, the proposed method fully explores the structural information of AST by combining three different traversal methods to obtain the spatial information of AST, which obtains more effective features than the traditional single traversal sequence. After these sequences are embedded, they are input into the transformer with deformable attention mechanism as three channels. The embedded network features and the formal processed features are weighted by the self-attention mechanism, then the fully connected network is used for binary classification.

In cross-version and cross-project scenarios, batch oversampling is used to solve the problem of unbalanced data sets and improve prediction performance. The experiments are conducted in 21 open source projects. The results showed that it has better performance than the state-of-the-art methods in most cases. The main contributions of this paper are as follows.

- Proposing triple traversal sequences for AST representation, convert the spatial information of AST into token sequences, and fully represent the features of AST.
- The Transformer with a deformable attention mechanism is used to process the word embedding matrix to discover defect patterns of different scales.
- The weights of semantic features and network features are determined through the self-attention mechanism to predict defects in files.

## Related work

### Software defect prediction

Software defects refer to statements, modules, or structures within the software that cause the software’s functions to operate abnormally, affecting its performance or efficiency. Defect prediction can help engineers assess the software condition during the early stages of the software lifecycle, thereby avoiding increases in costs and workload. However, due to the relatively fewer defects compared to normal samples, the sample class is extremely unbalanced, and some software modules have a limited number and a dispersion of defect patterns, which increases the difficulty of prediction. Moreover, cross-version and cross-project predictions are often more challenging, as different coding habits and software positioning pose greater challenges to the accuracy of defect prediction.

The software defect prediction model consists of several components, including feature acquisition, training, prediction, and evaluation. Early work mainly focused on feature selection and search algorithms, during which software metrics were used as input, and traditional machine learning served as the prediction model. It was not until the rise of deep learning that research began to favor feature extraction techniques for processing source code. At the same time, the learning models transitioned from machine learning to deep learning, such as convolutional neural network(CNN), long short-term memory network(LSTM), and Transformer. Several studies using deep learning have indicated that their performance, after evaluation, surpasses that of traditional machine learning [7].

### Multiple features prediction

In recent years, software defect prediction methods have shifted from single features to multiple features. And feature types have also changed from traditional metrics to semantic features and network features.

Shi et al. [8] extracted features from AST and software networks. Similar to most studies, they only used three types of AST nodes, with semantic features extracted by CNN and structural features extracted by skip-gram, and the two features were directly concatenated and input to classifier. Cross-version experiments on six Java datasets and four models showed that the hybrid features achieved a higher F1 score than traditional hand-crafted features or single semantic features or structural features. Average F1 reaching 0.560.

Wang et al. [9] proposed a method based on a gated hierarchical long short-term memory network (gh-LSTM). This method uses LSTM to extract features from word embeddings of AST and traditional metrics. The two features are then connected through a gated fusion layer for defect prediction. The results show that the average F1 is 0.612. It is higher than that of other methods. Tao et al. [10] used BiLSTM to extract semantic information from sequences of AST tokens and code change tokens. They also connected semantic features with traditional metrics and used a gated fusion mechanism to determine the combined ratio of the two features. Cross-version results showed the average F1 and MCC of the method reached 0.633 and 0.399. Their work indicate that multi-feature fusion has certain advantages.

As a contrast, Cui et al. [11] used metrics as network node attributes, established a complex software network, and used a group detection algorithm to divide the graph into multiple subgraphs. They learned the representation vectors of nodes through an improved graph neural network model. Finally, they used the node representation vectors to classify software defects. However, they only made predictions within the project, and the performance improvement was small compared to other models. It indicates that the combination of network features and software metrics does not have significant advantages.

Yu et al. [12] built a deep learning model for defect prediction based on self-attention mechanism (DPSAM) to automatically extract semantic features and perform defect prediction. They parsed the source code and embedded the AST to integers through the multi-head attention for learning. Zhao et al. [13] combined AST and CFG by dataflow. They designed AGN4D (attention-based graph neural network for directed graphs) to extract contextual features from the directed graph. The features and local features are added together, then pooling and predicted by the classifier. Compared to many models, their average F1(0.54) and AUC(0.675) improved significantly. Both articles show that the semantic features have good performance within and across projects.

These multi-feature software defect predictions have shown that the SDP performance is affected to a certain extent by the type of features, feature extraction technology and fusion methods. Apart from the relatively small improvement in the combination of network features and metrics, the combinations of other features have certain advantages. Many studies used fewer types of AST node and had low efficiency in AST representation and extraction. Feature fusion is merely a simple addition or concatenation, leading to a loss of information in the feature space. So the proposed method introduces more node types and attributes, and a more efficient representation method has been designed. A deformable attention mechanism is employed for the rapid extraction of complex features. At the same time, spatial information is preserved during feature fusion. Consequently, the method increases feature robustness and improve model prediction performance.

## Methodology

**Fig 1:**
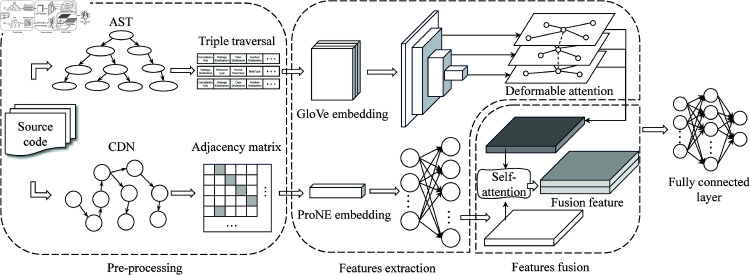
MFA overall framework.

Our MFA method consists of three main stages: source code preprocessing, feature processing, feature fusion and prediction. In the first stage, AST and CDN are constructed. In the second stage, glove and proNE methods are used to embed the parsed AST and CDN respectively. Semantic features are extracted through ResNet50 and deformable attention mechanism. The dimension of network features is expanded to match with semantic features. Finally, the self-attention mechanism is used to fuse features for defect prediction. The MFA framework is shown in Fig 1.

### Pre-processing

Pre-processing includes parsing the source code and representing its features. For semantic features, the source code is parsed into AST and traverse AST in three ways. For network features, CDN is built and represented by node pairs.

#### Triple traversal sequences of AST.

**Fig 2:**
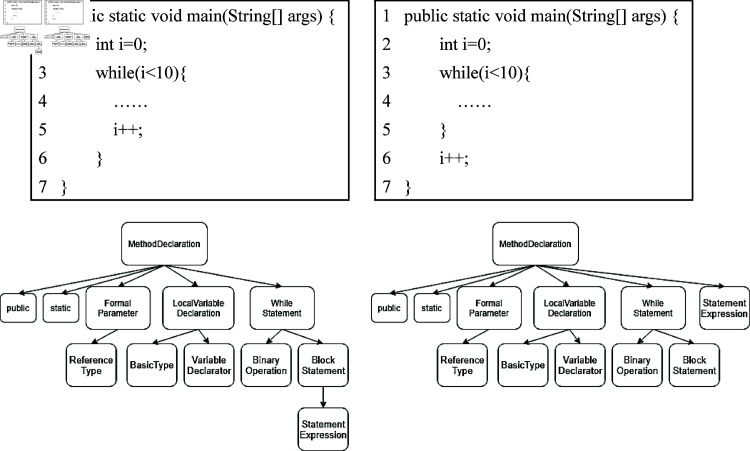
A similar code and its AST.

AST is generated by parsing the source code. In many researches [8–10,12], a single traversal is generally used to represent the AST tree. However, for different software structures, similar single traversal sequences will appear, resulting in a decrease in model resolution. As shown in Fig 2, left code is a correct program, while the right may fall into an infinite loop, but their single traversal sequences are the same because the “*i* + + ” fragment is at the end of the while loop. The AST of the program is shown below the code.

Therefore, we propose three AST traversal sequence methods, namely root-first, leaf-first and level-order traversal. Root-first is depth-first traversal, which starts from the root, walks to the leaf node of the path and then walks from the previous forked node to the leaf node of the path, and so on, until all nodes are visited. Leaf-first visits the leaf node first and then visits its root node. If the leaf node is not visited, its root node will never be visited. Level-order traversal is breadth-first traversal, which traverses according to the hierarchical structure of the tree. The latter two traversal methods will provide more details for single traversal, and they can be combined to represent a unique program structure.

**Table 1:** Triple traversal sequences of AST.

Traversal methods	Left code	Right code
root-first	MethodDeclaration,public,static,FormalParameter,	MethodDeclaration,public,static,FormalParameter,
	ReferenceType,LocalVariableDeclaration,BasicType,	ReferenceType,LocalVariableDeclaration,BasicType,
	VariableDeclarator,WhileStatement,BinaryOperation,	VariableDeclarator,WhileStatement,BinaryOperation,
	BlockStatement,**StatementExpression**	BlockStatement,**StatementExpression**
leaf-first	public,static,ReferenceType,FormalParameter,	public,static,ReferenceType,FormalParameter,
	BasicType,VariableDeclarator,LocalVariableDeclaration,	BasicType,VariableDeclarator,LocalVariableDeclaration,
	BinaryOperation,**StatementExpression**,BlockStatement,	BinaryOperation,BlockStatement,WhileStatement,
	WhileStatement,MethodDeclaration	**StatementExpression**,MethodDeclaration
level-order	MethodDeclaration,public,static,FormalParameter,	MethodDeclaration,public,static,FormalParameter
	LocalVariableDeclaration,WhileStatement,	LocalVariableDeclaration,WhileStatement,
	ReferenceType,BasicType,VariableDeclarator,	**StatementExpression**,ReferenceType,BasicType,
	BinaryOperation,BlockStatement,**StatementExpression**	VariableDeclarator,BinaryOperation,BlockStatement

The results of the three traversal of the program in Fig 2 are shown in Table 1. It can be seen from the table that the position of "StatementExpression" in the leaf-first or level-order has changed, indicating that they can distinguish the AST structure shown in Fig 2. But they still have their focus. Leaf-first gathers all leaf nodes together, which is similar to clustering operations, while level-order is better at hierarchical structures and has a better grasp of hierarchies.

For traversal types, include not only the AST definition node types but also the attributes under the nodes, such as method types and modifiers, but remove some elements that cannot be transferred for learning, such as method names and package names. They will cause a burden to identify defects in cross-versions or cross-projects scenario.

#### Class dependency network.

**Fig 3:**
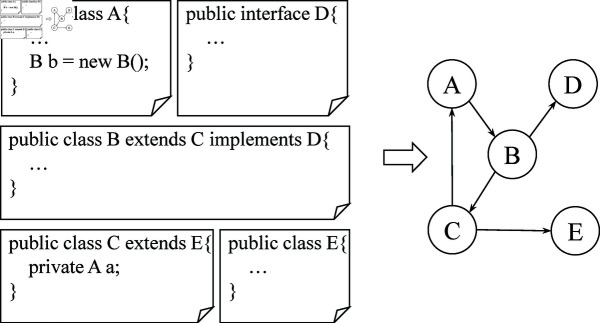
Class dependency network.

Java is an object-oriented programming language and the software written in it is based on classes. There are relationships between classes, such as references, object creation, inheritance and interface implementation, which are related to the software structure and function. Therefore, a class can be regarded as a node. As shown in Fig 3, class A calls class B, class B inherits C and implement interface D, and C not only inherits E, but also references A. The complex relationship between them can be converted into a corresponding graph. The dependencies between these classes are converted into the corresponding directed edges. The directly dependent classes are extracted in each file, and different classes are denoted as corresponding integers. Each class has a unique corresponding integer. Then, the edges between classes are recorded with integer pairs to prepare for network embedding.

### Features processing

Features process includes two parts: feature embedding and feature extraction. Among them, semantic feature extraction is particularly important. Since the pattern of the triple traversal sequences is more complex, professional deep learning is required to process it to obtain defect features.

#### Features embedding.

For semantic feature, Global Vectors for Word Representation(Glove) [14], an unsupervised learning algorithm is used to obtain vector representations of words. Training is based on the global word-word co-occurrence matrix of the corpus, and the generated vectors show a linear substructure of the word space. The embedding principle is shown in Eq 1 and Eq 2.


$$\begin{array}{ccc} J & =\overset{V}{\underset{i,j=1}{\sum }}f(X_{ij}){\left(w_{i}^{T}{\tilde{w}}_{j}+b_{i}+{\tilde{b}}_{j}-logX_{ij}\right)}^{2} & \end{array}$$ (1)

$$\begin{array}{ccc} f(x) & =\{\begin{array}{cc} {\left(\frac{x}{x_{max}}\right)}^{\alpha } & if\;x<x_{max}\\ 1 & otherwise \end{array} & \end{array}$$ (2)

Where V is the size of the vocabulary, f(x) is the weight function, $X_{ij}$ represents the number of times j appears in the context of i. $w_{i}$, ${\tilde{w}}_{j}$ both represent word vectors. $b_{i}$, ${\tilde{b}}_{j}$ are biases. *α* and $x_{max}$ are adjustable parameters of the weight function. *α* is 0.75 generally. $x_{max}$ has little impact on the performance of the model. It is fixed to 100 here.

As an unsupervised word embedding model, Glove requires more samples and data than supervised models. Therefore, in this method, the three sequences after traversing each file of the selected 21 projects are input into the word embedding model. The datasets include different projects and different versions.

For network feature, the ProNE network embedding method is involved. Network embedding is a technique that expresses network nodes or sub-networks in the form of vectors so that the model can be used directly. Among existing network embedding techniques, ProNE generally has better performance [15].

ProNE [16] expresses the network embedding as a sparse matrix factorization to effectively implement the initial node representation. Secondly, it uses high-order Cheeger inequalities to modulate the spectral space of the network and propagate the learned embedding in the modulated network, while integrating local smoothing and global clustering information.

#### Feature extraction.

Due to the complex and redundant structure of semantic features, defects are more weakly associated with them, and the defect structure is often scattered and irregular. However, deep learning technologies such as CNN and LSTM, which are good at learning spatial temporal regularities, have slow convergence and inaccuracy problems. Extremely unbalanced software samples increase the difficulty of defect prediction. Therefore, the deformable attention mechanism [17] is used to extract semantic features. This method relies on the transformer and its attention mechanism.

The attention mechanism can effectively capture the relationship between distant elements in a sequence without being restricted by the sequence length. This is why it is favored by researchers in this field, as the lengths of the AST vary, and the defect patterns require a comprehensive judgment of long-distance phrases.

Attention calculated by three vectors, named Query, Key and Value. The Query at each position is dot-producted with the Key at all positions to get an attention score. These scores are then used to weight the Value to generate the output for each position. This global calculation allows each position to connect directly to other positions. But the problem with normal attention mechanism is that they require a large number of samples to converge. This is fatal for software with a small sample size, and deformable attention solves this problem.

In the encoder stage, the value of each point in the feature vector is determined by a specific number of surrounding points, and the positions of these points are also learned. By assigning a small fixed number of keys to each query, the problems of convergence and feature space resolution can be alleviated.

Given an input feature map $x\in {\mathbb{R}}^{C\times H\times W}$, q represents query, then the feature of q is $z_{q}$, the reference point of q is $p_{q}$, and the deformable attention feature formula is


$$\begin{array}{ccc} DeformAttn(z_{q},p_{q},x)=\overset{M}{\underset{m=1}{\sum }}W_{m}[\overset{K}{\underset{k=1}{\sum }}A_{mqk}\cdot W_{m}^{\prime }x(p_{q}+△p_{mqk})] & & \end{array}$$ (3)

**Fig 4:**
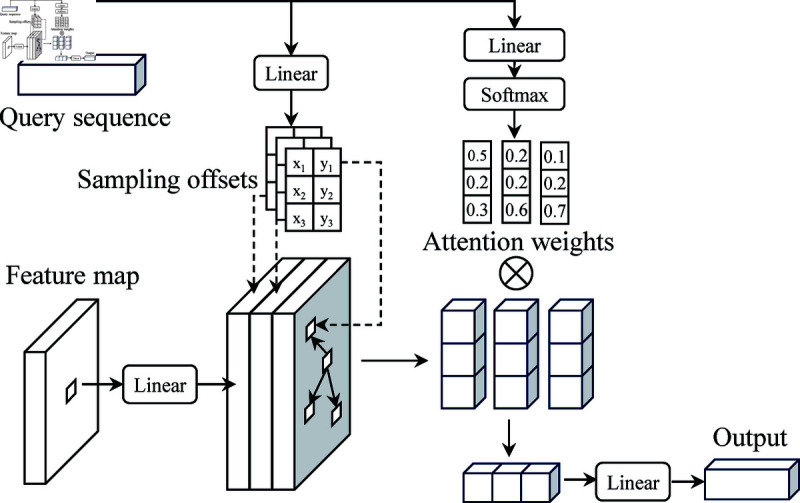
Deformable attention mechanism.

Where m, k and K represent the attention head, sampled key, and the total number of sampled keys. $W^{\prime }$ is the projection matrix of input value, and *W* is output projection matrix. $△p_{mqk}$ and $A_{mqk}$ represent the offset and attention weight of the kth sampling point of the mth attention head, respectively. Both $△p_{mqk}$ and $A_{mqk}$ are linear projections obtained from the query feature $z_{q}$. In this process, the query feature $z_{q}$ is linearly mapped, the sampling point offset $△p_{mqk}$ is determined by the floating point numbers in the xy direction, and the attention weight $A_{mqk}$ is obtained by $z_{q}$ after softmax. The principle is shown in Fig 4.

In the encoder stage, the calculation speed is related to the size of the feature space and the number of attention points, while in the decoder stage, the complexity is independent of the space, so a lot of calculation time is saved. After the semantic features pass through Resnet50, the outputs of the first, second, and third layers are used as the input of the Transformer, and attention is applied between the features of these different scale layers to enhance the performance of the features. At the same time, the output of the three decoders are input into the self-attention mechanism to participate in the weight allocation of the fusion features.

For embedded network features, CNN is used to expand its channels to make them consistent with the dimensions of semantic features to enhance their competitiveness, and then input them into the self-attention mechanism for feature fusion.

### Features fusion and prediction

After processing, the semantic features and network features have different emphases. The degree of association with defects is also different. Therefore, when fusion of features is performed, it is necessary to determine the weight distribution of different features without destroying the feature space structure.


$$\begin{array}{ccc} \begin{array}{ccc} M_{c}(F) & =\sigma (MLP(AvgPool(F))+MLP(MaxPool(F))) & \\ & =\sigma (W_{1}(W_{0}(F_{a}vg))+W_{1}(W_{0}(F_{m}ax))) \end{array} & & \end{array}$$ (4)

**Fig 5:**
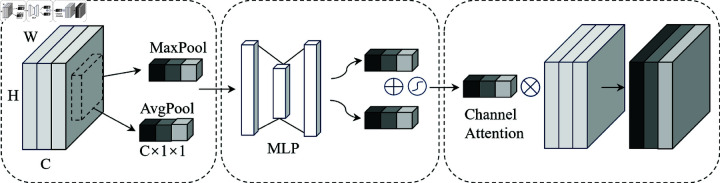
Channel self-attention.

To solve this problem, the channel attention in the Convolutional Block Attention Module(CBAM) [18] is introduced, as shown in Fig 5. CBAM determines the weight of each channel through channel convolution, pooling and multilayer perceptron training. Then assigns weights to different channels. When the weight is large, the value of the channel feature map increases accordingly. In contrary, the value of the channel feature map will be smaller, inhibiting its activation. Eq 4 shows the changes of the feature after passing through the CBAM attention module.

## Experiments

### Datasets

21 Java datasets from the PROMISE database are selected for experiments. According to statistics, Java-written software is the most widely used in defect prediction [1,2], providing more options for comparative experiments. AST is generated by Javalang, a normally used python package to parse the source code, and use a software named Class Dependency Analyzer to extract class dependency to construct CDN. According to the source code and dataset file names, most of the source code is parsed in the experiment, but some files still failed to parse due to not existing and syntax errors. Therefore, the experimental dataset is a simplified version of the original dataset, retaining all correct and searchable files. The details of the datasets are shown in Table 2.

**Table 2:** Datasets detail.

No.	Project	Original files	defect rates	Current files	defect rates
1	ant-1.4	178	22.47%	166	21.69%
2	ant-1.6	351	26.21%	350	26.29%
3	ant-1.7	745	22.28%	741	22.40%
4	camel-1.2	608	35.53%	595	36.30%
5	camel-1.4	872	16.63%	844	17.18%
6	camel-1.6	965	19.48%	921	20.41%
7	jedit-3.2	272	33.09%	248	33.87%
8	jedit-4.0	306	24.51%	281	23.84%
9	jedit-4.1	312	25.32%	287	23.69%
10	lucene-2.0	195	46.67%	186	48.92%
11	lucene-2.2	247	58.30%	234	61.11%
12	lucene-2.4	340	59.71%	329	61.70%
13	poi-1.5	237	59.49%	235	60.00%
14	poi-2.5	385	64.42%	380	65.26%
15	poi-3.0	442	63.57%	438	64.16%
16	velocity-1.4	196	75.00%	192	75.52%
17	velocity-1.5	214	66.36%	214	66.36%
18	velocity-1.6	229	34.06%	229	34.06%
19	xalan-2.4	723	15.21%	668	16.02%
20	xalan-2.5	803	48.19%	754	50.27%
21	xalan-2.6	885	46.44%	875	46.97%

### Settings

This model is programmed in the environment of Python 3.9, PyTorch 2.0.1 and Cuda 11.8. The experiment is carried out in two scenarios. In the cross-version scenario, the training set and the test set come from different versions of the same software. The training set is the version with a smaller version number, while the test set is the version with a larger version number. The old version can be used to predict defects in the new version. For example, ant1.4 is used to predict defects in ant1.6, and ant1.6 is used to predict defects in ant1.7.

In the cross-project scenario, all versions of a project are used as training sets in the experiment, and all versions of other projects are used as test sets for prediction. For example, ant1.4, ant1.6, and ant1.7 are merged into one training set, camel, jedit, lucene, poi, velocity, and xalan are used as test sets. The integration of multiple versions and projects is conducive to enriching the types of code structures.

Since the dataset for software defect prediction is class-imbalanced, batch oversampling is performed to balance the dataset. Batch oversampling can determine the ratio of positive and negative examples in each batch of training sets according to the set sampling ratio. The hyperparameters in the experiment are shown in Table 3.

**Fig 6:**
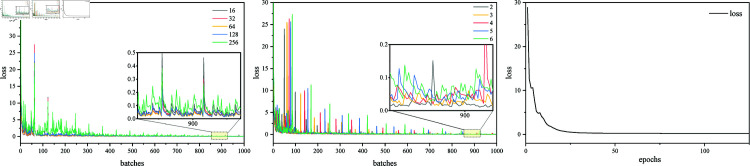
Loss of Glove in different settings.

A total of 6,109 words were used for word embedding. To determine the embedding dimensions and window sizes, dimensions of 16, 32, 64, 128, 256 were set, along with window sizes of 2, 3, 4, 5, 6, resulting in five groups of experiments. According to Fig 6, the loss for the first 1,000 batches shows that the loss is lowest when the embedding dimension is 64. Additionally, the embedding window at sizes 2 or 3 achieves better results, with size 3 being more stable to a certain extent. Under the condition of selecting the first two parameters, experiments conducted over 125 epochs indicate that the model essentially converges after 75 epochs. To be cautious, 100 epochs were chosen. For Transformer, retaining the original optimizer and learning rate yields better results in experiments. When the encoder and decoder exceed three, there is no improvement in the results; instead, it increases computation time. The model can converge in no more than 20 epochs during training.

**Table 3:** Hyperparameter settings.

Parameters	Values
Embedding dimension	64
Glove window	3
Glove epoch	100
Optimizer	AdamW
Learning rate	0.001
Training epoch	20
Encoder number	3
Decoder number	3

### Baseline models

BiLSTM: Dam et al. [19] input AST sequence into LSTM for word embedding, then input the embedding vector into LSTM to extract syntax and semantics, and finally predict through classifier.

N2D: Qu et al. [20] use skip-gram to embed the sequence of CDN traversal, then combine the embedding features with traditional metrics and predict through classifier.

GCN: Zeng et al. [21] use node2vec network embedding and use traditional software code features as attributes of CDN nodes. Finally, CDN is input into GCN to obtain deep features and then classified.

CGCN: Zhou et al. [22] transform source code into AST and CDN. The integer vector of AST is input into CNN to capture semantic information, and the graph convolutional network (GCN) learns the structural information of CDN. The learned deep features are connected with traditional metrics and the classifier is trained to achieve more accurate defect prediction.

### Evaluation metrics

Five common evaluation metrics are used to evaluate the performance of the prediction model, including accuracy, precision, recall, F1, and area under curve(AUC).


$$\begin{array}{ccc} acc & =\frac{TN+TP}{TN+TP+FN+FP} & \end{array}$$ (5)

$$\begin{array}{ccc} precision & =\frac{TP}{TP+FP} & \end{array}$$ (6)

$$\begin{array}{ccc} recall & =\frac{TP}{TP+FN} & \end{array}$$ (7)

$$\begin{array}{ccc} F1 & =\frac{2*precision*recall}{precision+recall} & \end{array}$$ (8)

TP, TN, FN and FP come from the confusion matrix which is a tool used to evaluate the performance of classification models. Defect samples are considered positive. “T” denotes the prediction is correct and "P" denotes the samples are predicted as positive(defect). “F” denotes the prediction is false and “N” denotes the samples are predicted as negative(non-defect). For example, TP represents the number of actual defect samples predicted as defective.

AUC is the area under the receiver operating characteristic curve(ROC). The x-axis of ROC is the ratio of non-defective modules classified as defective(False Positive Rate, FPR). The y-axis is Recall. It represents the corresponding FPR and Recall values under different thresholds. The threshold is determined by the probability that different samples are predicted as defects. The closer the AUC value is to 1, the better the classifier’s discrimination ability is.

## Results

Table 4 is the results of the cross-version experiment. The first two columns represent the training set and the test set respectively, and the last column is the sampling ratio, which indicates the proportion of defect-free samples in each batch. The results show that the average ACC of all cross-version experiments is 0.7, the average F1 value is 0.614, and the average AUC is 0.711, achieving good prediction performance. However, camel1.4-1.6 has the lowest F1 and AUC. The ACC value of velocity1.4-1.5 is the lowest among all cross-versions, followed by xalan2.4-2.5. The defect rates of these versions are at two extremes, either too high or too low. Oversampling cannot completely solve this problem, because duplicate samples may be sampled, resulting in a decrease in the model’s discriminative ability. The average sampling ratio is 0.468, indicating that most projects have better performance when close to 0.5, and balancing different types of samples is conducive to performance improvement.

**Table 4:** Cross-version results.

No.	Project	Source	Target	Acc	Precision	Recall	F1	AUC	Ratio
1	ant	1.4	1.6	0.809	0.629	0.663	0.646	0.775	0.45
2	ant	1.6	1.7	0.777	0.502	0.657	0.569	0.814	0.5
3	camel	1.2	1.4	0.678	0.294	0.628	0.401	0.716	0.5
4	camel	1.4	1.6	0.733	0.335	0.314	0.324	0.562	0.6
5	jedit	3.2	4.0	0.769	0.511	0.672	0.581	0.767	0.55
6	jedit	4.0	4.1	0.787	0.538	0.721	0.616	0.832	0.35
7	lucene	2.0	2.2	0.624	0.796	0.517	0.627	0.684	0.6
8	lucene	2.2	2.4	0.635	0.790	0.557	0.653	0.679	0.55
9	poi	1.5	2.5	0.729	0.727	0.935	0.818	0.674	0.35
10	poi	2.5	3.0	0.744	0.738	0.932	0.824	0.721	0.35
11	velocity	1.4	1.5	0.556	0.737	0.514	0.606	0.610	0.45
12	velocity	1.5	1.6	0.651	0.492	0.808	0.612	0.717	0.55
13	xalan	2.4	2.5	0.586	0.585	0.609	0.597	0.631	0.3
14	xalan	2.5	2.6	0.718	0.672	0.781	0.722	0.769	0.45
15	Avg			0.700	0.596	0.665	0.614	0.711	0.468

**Table 5:** Cross-project results.

No.	Source	Target	Acc	Precision	Recall	F1	AUC	Ratio
1	camel	ant	0.744	0.413	0.235	0.300	0.568	0.4
2	jedit	ant	0.787	0.607	0.584	0.595	0.786	0.55
3	lucene	ant	0.638	0.780	0.529	0.630	0.697	0.35
4	poi	ant	0.653	0.763	0.660	0.708	0.687	0.3
5	velocity	ant	0.559	0.676	0.447	0.538	0.619	0.35
6	xalan	ant	0.658	0.557	0.606	0.581	0.694	0.4
7	ant	camel	0.778	0.521	0.619	0.566	0.791	0.55
8	jedit	camel	0.733	0.502	0.571	0.534	0.774	0.6
9	lucene	camel	0.571	0.774	0.375	0.505	0.670	0.6
10	poi	camel	0.606	0.803	0.504	0.620	0.713	0.55
11	velocity	camel	0.572	0.672	0.499	0.572	0.628	0.45
12	xalan	camel	0.625	0.516	0.644	0.573	0.652	0.5
13	ant	jedit	0.790	0.548	0.582	0.564	0.799	0.55
14	camel	jedit	0.749	0.426	0.226	0.295	0.613	0.4
15	lucene	jedit	0.642	0.722	0.629	0.672	0.701	0.3
16	poi	jedit	0.726	0.726	0.916	0.810	0.676	0.35
17	velocity	jedit	0.627	0.695	0.625	0.658	0.655	0.45
18	xalan	jedit	0.680	0.620	0.467	0.533	0.704	0.6
19	ant	lucene	0.790	0.547	0.588	0.567	0.746	0.55
20	camel	lucene	0.717	0.372	0.317	0.342	0.600	0.45
21	jedit	lucene	0.787	0.607	0.584	0.595	0.789	0.6
22	poi	lucene	0.721	0.716	0.930	0.809	0.694	0.3
23	velocity	lucene	0.628	0.660	0.729	0.693	0.629	0.35
24	xalan	lucene	0.681	0.628	0.450	0.525	0.694	0.55
25	ant	poi	0.730	0.447	0.663	0.534	0.760	0.55
26	camel	poi	0.662	0.338	0.470	0.393	0.624	0.5
27	jedit	poi	0.614	0.399	0.868	0.547	0.800	0.4
28	lucene	poi	0.650	0.711	0.675	0.692	0.697	0.4
29	velocity	poi	0.625	0.658	0.723	0.689	0.646	0.3
30	xalan	poi	0.657	0.555	0.609	0.581	0.695	0.6
31	ant	velocity	0.798	0.618	0.357	0.453	0.756	0.55
32	camel	velocity	0.744	0.413	0.235	0.300	0.632	0.5
33	jedit	velocity	0.733	0.502	0.676	0.576	0.748	0.55
34	lucene	velocity	0.657	0.662	0.840	0.741	0.691	0.35
35	poi	velocity	0.736	0.762	0.851	0.804	0.746	0.45
36	xalan	velocity	0.658	0.557	0.606	0.581	0.656	0.55
37	ant	xalan	0.728	0.451	0.755	0.565	0.798	0.5
38	camel	xalan	0.717	0.372	0.317	0.342	0.579	0.3
39	jedit	xalan	0.784	0.614	0.530	0.569	0.790	0.6
40	lucene	xalan	0.637	0.796	0.508	0.620	0.713	0.45
41	poi	xalan	0.629	0.770	0.594	0.671	0.704	0.45
42	velocity	xalan	0.628	0.660	0.729	0.693	0.610	0.35
43	Avg		0.687	0.598	0.579	0.575	0.696	0.462

Table 5 shows the results of the cross-project experiment. The first two columns show the training set and the test set, and the last column shows the ratio of defect-free samples in each batch of the training set. The average ACC is 0.687, the average F1 is 0.575, and the average AUC is 0.696. In all projects, camel has poor results as the target, and velocity has poor results as the training set. This is related to the defect rate of the dataset and the file structure and length of the two projects. This may be due to the purpose and background of the software. Further analysis is made in the comparative experiment.

### Cross-version comparison experiment

**Fig 7:**
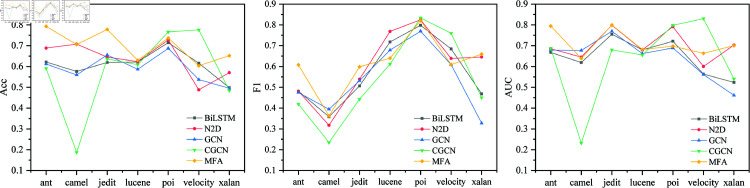
Cross-version comparison experiment results.

The cross-version comparison experiment is shown in the Fig 7. Compared with other models, MFA has the highest average ACC, F1, and AUC. This shows that MFA has certain advantages in cross-version scenarios. In the prediction of projects such as ant and jedit, it shows high performance. The Acc value of Ant1.4-1.6 prediction reach 0.809, the F1 value of poi2.5-3.0 is 0.824, and the AUC of jedit4.0-4.1 prediction is 0.832, which shows that the model has high performance and has strong recognition ability for defect patterns of complex traversal sequences.

**Fig 8:**
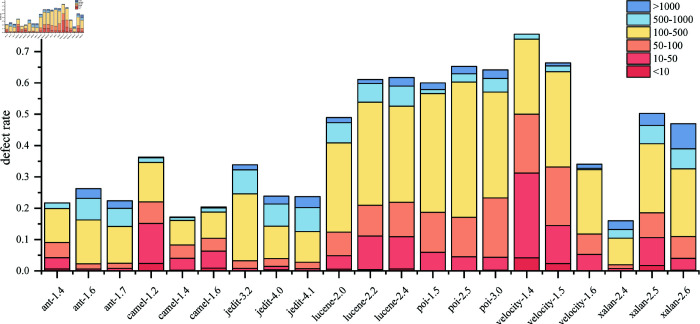
Defect rate of different sequence lengths in each version.

However, the results of camel1.4-1.6, velocity1.4-1.5, and xalan2.4-2.5 performed poorly compared to other models. The reasons are different. There are a large number of small file defects (AST sequence length is less than 50) in velocity1.4 and 1.5. As shown in Fig 8, in deep learning, small file defects are prone to losing details during downsampling, resulting in a decline in model performance. In camel1.4 and xalan2.4, the defect rate is too low and there are fewer defective samples, resulting in a decline in model performance.

### Cross-project comparison experiment

**Fig 9:**
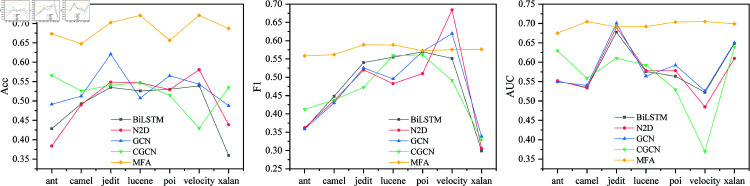
Cross-project comparison experiment results.

The cross-project comparison results are shown in Fig 9. The average ACC, F1, and AUC of MFA all exceed those of other models, which are 0.687, 0.575, and 0.696, respectively. All versions of a project as a dataset for experiments, which is conducive to enriching the file structure and defect types in the dataset and increasing the number of cross-project samples, which is important for deep learning. The highest Acc, F1, and AUC are 0.798, 0.810, and 0.8, respectively, indicating that MFA has higher performance.

**Fig 10:**
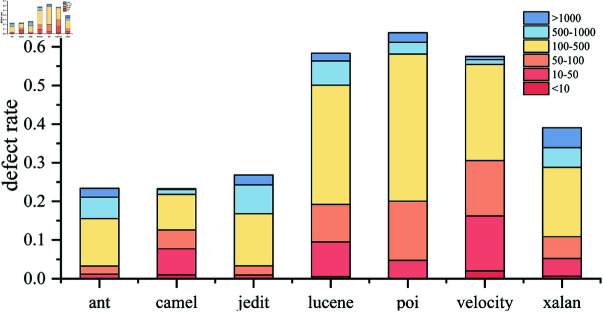
Defect rate of different sequence lengths in each project.

Among all cross-project predictions, camel’s prediction results are the worst, which is related to its data set. As can be seen from Fig 10, its small file (short sequence) defect ratio is large and the defect rate is small, which means that the quality and quantity of defect samples are insufficient. In feature extraction, since long and short files cannot be taken into account at the same time, small file feature extraction is insufficient, which in turn affects cross-project performance. When ant and jedit are used as training sets for cross-project prediction, the performance is often better, which is related to their lower short sequence defect ratio. In Fig 10, the defect ratio of files less than 50 in ant and jedit is the lowest compared to other projects. Because small files do not have enough defect patterns, the model has poor learning ability for them.

**Fig 11:**
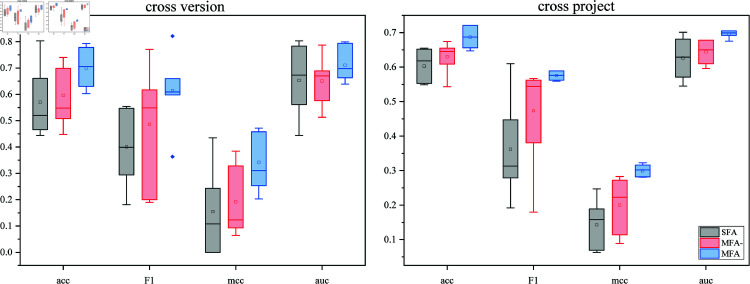
Ablation experiments.

## Discussion

In cross-version experiments, the datasets where MFA did not perform well are velocity, lucene, and poi. In particular, velocity is not the best in terms of Acc, F1, and AUC. It has been mentioned that its defect files mostly consist of small files in Fig 8. Although for adapting varying sample lengths, the padding length for each batch is minimized as much as possible, the longest sequence still reached 6721. Without truncating long sequences, downsampling must be employed. However, this inevitably leads to the loss of some details in the features of short sequences before deformable attention be performed. In velocity, more than half of the defect sequence lengths are less than 100, which affects the training effectiveness. The advantage shown by CGCN on velocity stems from CNN’s tolerance for short sequences.

The advantages of MFA are evident in cross-project tasks. It leads in almost all evaluation metrics and has stable performance across each project. This is attributed to the handling of long sequences, where the triple traversal sequences of AST enhance the distinction between files and deformable attention allowing for faster convergence with a limited number of samples. The GPU resources used throughout the process do not exceed 4G; however, the efficiency of deformable attention is significantly affected by the size of the input matrix. The MFA model has an overall parameter size of 25.4M, compared to the representative AST model, which is more than the TBCNN [23](0.5M) but less than code2seq [24](61M). However, it can achieve similar performance, with code2seq achieving an F1 of 0.592 across projects in large Java datasets.

To demonstrate the role of semantic embedding and network features, ablation experiments are carried out in two scenarios: cross-version and cross-project, and four evaluation metrics are used to represent the model performance, as shown in Fig 11. In addition, the Matthews Correlation Coefficient(MCC) is involved. MCC is more comprehensive and less biased than F1 [25]. MFA is the proposed model, MFA- represents a multi-feature model without glove word embedding, and SFA represents a model with pure semantic features and no glove.


$$\begin{array}{ccc} MCC=\frac{TP\times TN-FP\times FN}{\sqrt{\left(TP+FP)(TP+FN)(TN+FP)(TN+FN\right)}} & & \end{array}$$ (9)

In both scenarios, the four metrics of MFA perform better than the other two models. After adding network features and glove word embedding, the performance of semantic features gradually improves, indicating that the addition of the two modules is effective. Overall, the average performance of MFA-compared with SFA with the addition of network features has increased. This shows that the addition of network features is a good supplement to semantic features, which helps to improve the stability of overall prediction, but its improvement is limited. On individual data sets, such as jedit cross-version and velocity cross-project, the addition of network features reduces performance. After adding glove, the lower limit of cross-project performance increases more, reflecting its advantages. Word embedding provides more semantic information and increases the stability of semantic features.

From the perspective of MCC alone, semantic features show instability. Fusion with network features can obviously present more concentrated results, and glove further improves the performance of semantic features. However, the F1 value seems to be biased in the evaluation. F1 has abnormal points in cross-versions, and its fluctuation is greater than other metrics. Therefore, F1 may be unreliable in single-project evaluation, but the average F1 can reflect the performance of the model.

## Limitation

AST nodes selection: In the experiment, custom nodes such as function names and operators are removed to ensure the transferability of models across versions and projects. This may cause the loss of some details of the AST, thus affecting the model’s capture of defective modules. In the future, we will try to retain all AST nodes and simplify similar nodes.

Batch oversampling setting: In the experiment, due to the different characteristics of each dataset, only through experiments can be known how its performance is under the specific ratio. Therefore, a step of 0.05 between 0.05-0.9 is used and 19 experiments are conducted for each dataset, which affected the experimental efficiency. In the future, we will use the sampling ratio of batch oversampling as a learnable variable to reduce unnecessary experiments.

Sequence length padding: In the experiment, padding is reduced as much as possible, and the file length is shortened to increase the training speed. However, since the lengths of files in each project vary, during training, all sequences are padded with 0 to the longest AST sequence in the training set and test set, so that all sequences are input into the model with the same length. This consumes a lot of space, causing the details of short sequence files to be lost during feature extraction, which affects the performance of the model to a certain extent. In the future, we will consider using different models to extract short and long sequences and finally make a comprehensive prediction.

## Conclusion

In this research, a software defect prediction model MFA based on the attention mechanism is proposed. This model makes full use of the structural and semantic information of AST and CDN. After the two features are embedded, they are extracted and fused by the attention mechanism for defect prediction. The experimental results show that the average ACC, F1, AUC of MFA in the cross-version scheme reach 0.7, 0.614 and 0.711. And the average ACC, F1, AUC in the cross-project scheme are 0.687, 0.575 and 0.696, having more advantages than the LSTM model based on a single feature and the GNN model based on multiple features, indicating that this method has better prediction performance by utilizing attention mechanism to capture subtle defect patterns in semantic and network features. The ablation experiment also shows that network features and semantic features can complement each other and jointly improve the performance of software defect prediction. There is still some work worth further study on this method, such as the full representation of source code information, the unification of sequence length and cross-language prediction.
